# Supplementary material for: Targeted mechanical stimulation via magnetic nanoparticles guides in vitro tissue development
Source: Nat Commun. 2023 Aug 30;14:5281. doi: 10.1038/s41467-023-41037-8 (PMC10465512; doi:10.1038/s41467-023-41037-8)
Supplement: Supplementary file 1 — Supplementary Information [file 41467_2023_41037_MOESM1_ESM.pdf]

Targeted mechanical stimulation via magnetic nanoparticles guides in-vitro tissue development

Abdel Rahman Abdel Fattah[1*], Niko Kolaitis[1], Katrien Van Daele[1], Brian Daza[1], Andika Gregorius Rustandi[1], Adrian Ranga[1*]

[1] Laboratory of Bioengineering and Morphogenesis, Biomechanics Section, Department of Mechanical Engineering, KU Leuven, Leuven, Belgium

* email: adrian.ranga@kuleuven.be, aabdelfattah@cemm.oeaw.ac.at

Supplementary Figures

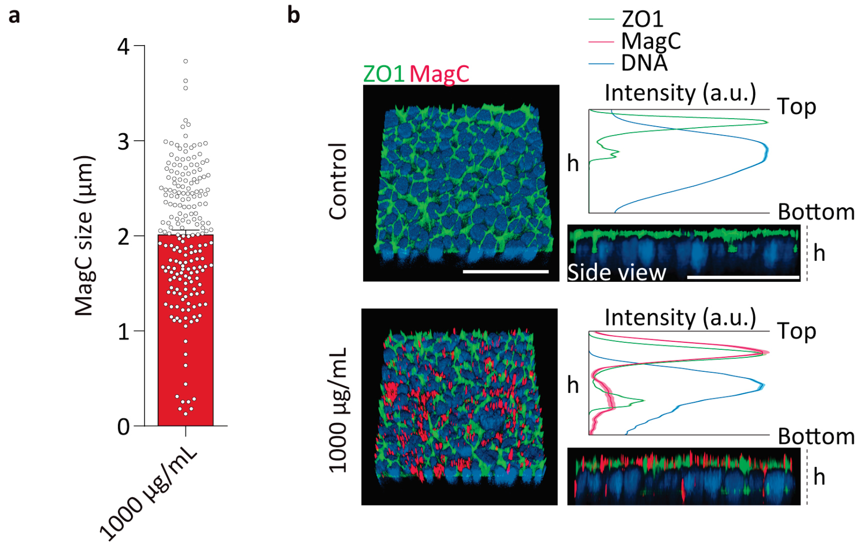

Supplementary Fig.1: MagC size and location during hPSC magnetization. a MagC sizes after fluorescent tagging (n = 3 independent experiments for 203 MagCs). **b** 3-D reconstructed representative images showing ZO1 and MagC localization in control and MagC (1,000 µg/mL) conditions (n = 3 independent experiments). Profiles showing ZO1, MagC and nuclei vertical position (n = 3 independent experiments for a total of 50 profiles). Scalebar 50 µm. Error bars are SEM. Source data are provided as a Source Data file.

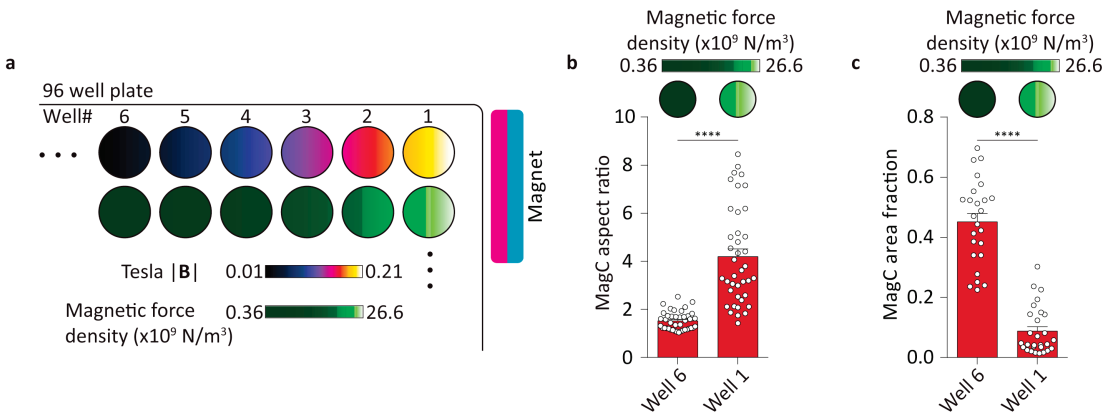

Supplementary Fig.2: Effect of distance-from-magnet on magnetic force magnitude and MagC geometry. a Magnetic field and force densities in wells of a 96 well-plate at various distances from a magnet surface as simulated using a 30 x 30 x 15 mm N45 permanent magnet. **b** MagC aspect ratio of day 11 hNTmOs in wells of a 96 well-plate at close (well 1) and far (well 6) distances from the magnet surface (n = 3 independent experiments for 41 (well 6) and 40 (well 1) hNTmOs). Statistical analysis was determined by unpaired two-sided t-test where * represents *p*-value for well 6-well 1 $p = 2.01022 \times 10^{-12}$. **c** MagC area ratio of day 1 and 11 hNTmOs in wells of a 96 well-plate at close (well 1) and far (well 6) distances from the magnet surface (n = 3 independent experiments for25 (well 6) and 28 (well 1) hNTmOs). Statistical analysis was determined by unpaired two-sided t-test where * represents *p*-value for well 6-well 1 $p = 3.67723 \times 10^{-16}$. Error bars are SEM. Source data are provided as a Source Data file.

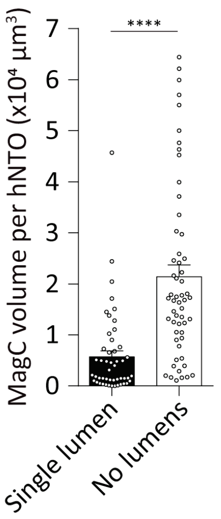

Supplementary Fig.3: Effect of MagC volume on lumen generation. Estimation of MagC volumes in day 11 hNTmOs with single or no lumen formation (n = 3 independent experiments for 51 (single lumen) and 56 (no lumens) hNTmOs). Statistical analysis was determined by unpaired two-sided t-test where * represents *p*-value for single lumen-no lumen $p = 4.54107 \times 10^{-8}$. Error bars are SEM. Source data are provided as a Source Data file.

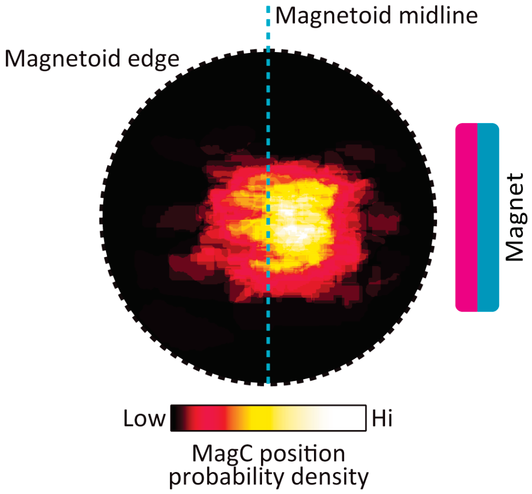

Supplementary Fig.4: MagC localization within magnetoids. Dimensionless analysis of MagC position probability density in magnetoids (n = 4 independent experiments, data from **Fig.4a**).

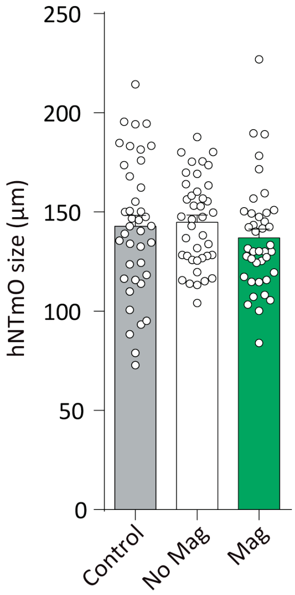

Supplementary Fig.5: Magnetoids do not exhibit a size change. Day 11 hNTmO sizes in various conditions (n = 3 independent experiments for a total of 40 hNTmOs per condition). Error bars are SEM. Source data are provided as a Source Data file.

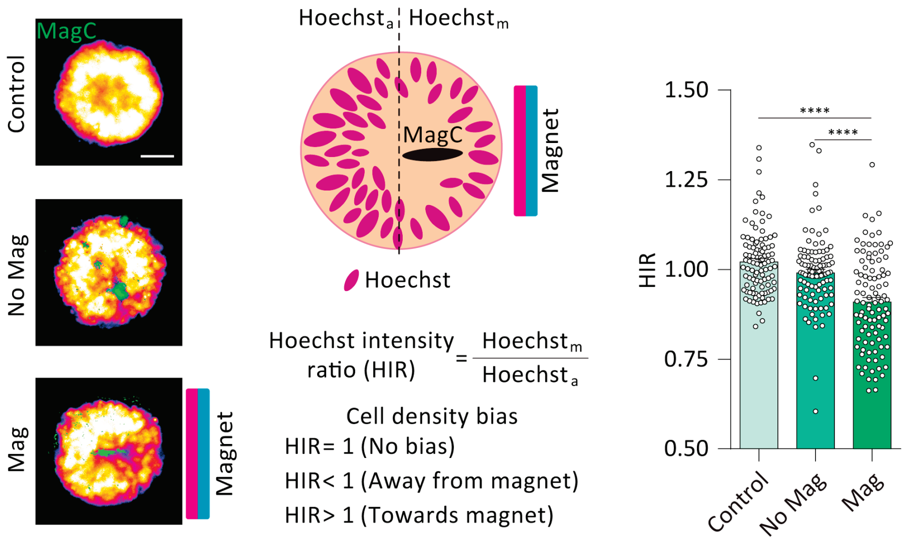

Supplementary Fig.6: Local changes of cell density in actuated magnetoids. Representative images for Hoechst staining of day 11 hNTmOs under various actuation conditions (n = 4 independent experiments from Figures 4 and 5). Schematic representation of biased cell density in hNTmO under the influence of magnetic field. Hoechst intensity ratio of day 11 hNTmOs for various actuation conditions (n = 4 independent experiments from Figures 4 and 5 for a total of 100 organoids per condition). Statistical analysis was determined by unpaired two-sided t-test where * represents *p*-values for Control-Mag $p = 3.39521 \times 10^{-11}$, No Mag-Mag $p = 6.27307 \times 10^{-5}$. Scalebar 50 µm. Error bars are SEM. Source data are provided as a Source Data file.

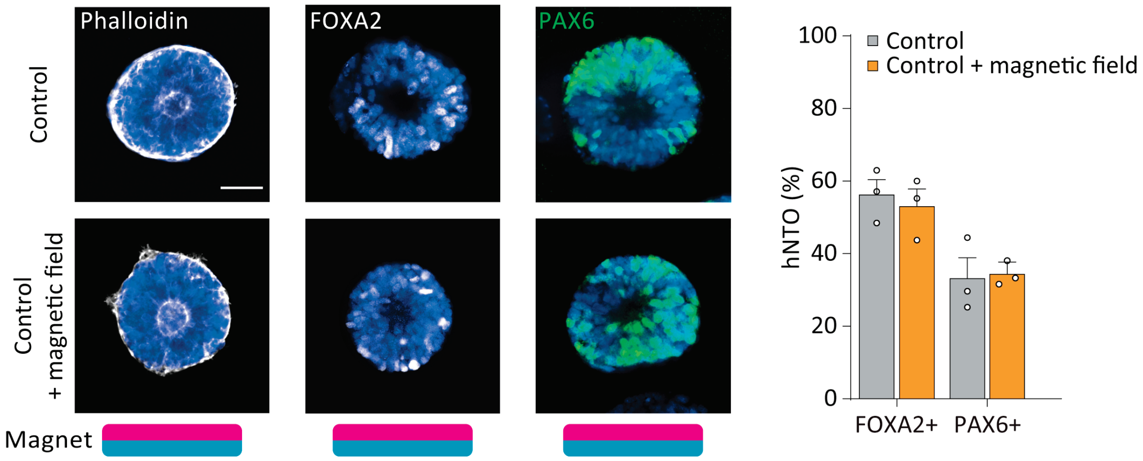

Supplementary Fig.7: Magnetic field has negligible effect on FOXA2 and PAX6 induction. Representative images phalloidin, FOXA2 and PAX6 expressions in day 11 MagC-free hNTOs under control and magnetic conditions (n = 3 independent experiments). Induction rates of FOXA2 and PAX6 in hNTOs cultured under control and magnetic conditions (n = 3 independent experiments for a total of 90 hNTOs per condition). Scalebar 50 µm. Error bars are SEM. Source data are provided as a Source Data file.

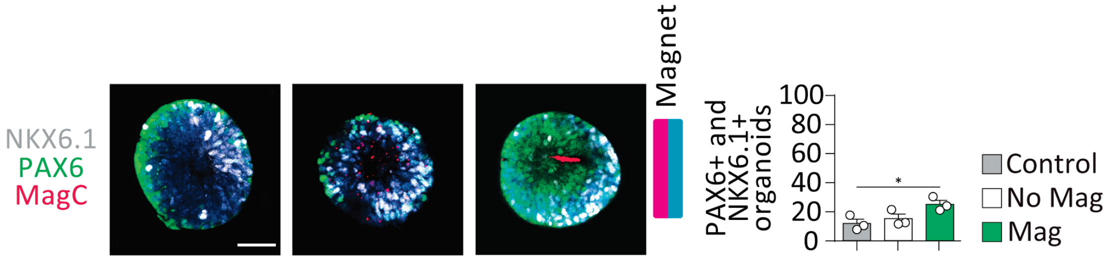

Supplementary Fig.8: Effect of local actuation on frequency of double (dorsal and ventral) positive organoids. Representative images for ventral (NKX6.1) and intermediate (PAX6) double positive day 11 organoids under various actuation conditions (n = 3 independent experiments from **Fig. 5a**). Quantification of double positive organoids for various actuation conditions (n = 3 independent experiments from **Fig.5a** for a total of >60 organoids per condition). Statistical analysis was determined by unpaired two-sided t-test where * represents *p*-value for Control-Mag $p = 0.0308$. Scalebar 50 µm. Error bars are SEM. Source data are provided as a Source Data file.

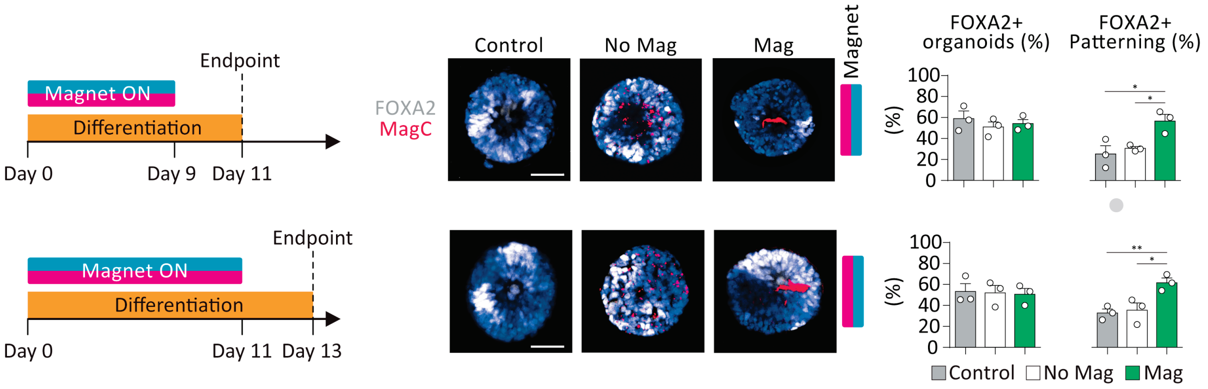

Supplementary Fig.9: Local actuation forms stable (up to two days) patterns after magnetic field removal. Schematic representation of the experimental timeline. Representative images showing Day 11 and Day 13 organoids with MagC positions and FOXA2 expressions in different conditions (n = 3 independent experiments). Percentages of FOXA2+ organoids and patterning in FOXA2+ organoids in different conditions (n = 3 independent experiments for 73 (Day 11 control), 131 (Day 11 no mag), 93 (Day 11 mag), 77 (Day 13 control), 91 (Day 13 no mag), and 71 (Day 13 mag) hNTmOs). Statistical analysis was determined by unpaired two-sided t-test where * represents *p*-values for Day 11 Control-Mag $p = 0.0350$, Day 11 No Mag-Mag $p = 0.0158$, Day 13 Control-Mag $p = 0.0077$, Day 13 No Mag-Mag $p = 0.0338$. Scalebar 50 µm. Error bars are SEM. Source data are provided as a Source Data file.

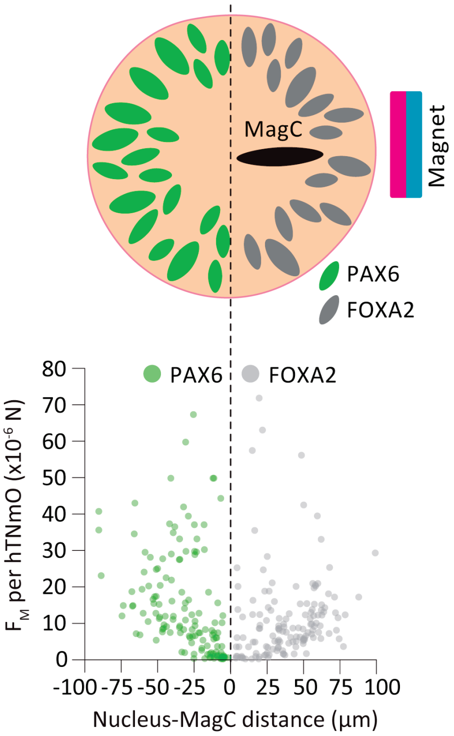

Supplementary Fig.10: Larger forces from more expansive domains. Schematic representation of biased FOXA2 and PAX6 expressions under magnetic actuation condition. Correlation of the magnetic force generated by a MagC and the distance separating the MagC and the nucleus expressing FOXA2 or PAX6 fates (n = 4 independent experiments, data from **Fig.5a**, for a total of 140 organoids per fate). Source data are provided as a Source Data file.
