# Supplementary material for: Targeted mechanical stimulation via magnetic nanoparticles guides in vitro tissue development
Source: Nat Commun. 2023 Aug 30;14:5281. doi: 10.1038/s41467-023-41037-8 (PMC10465512; doi:10.1038/s41467-023-41037-8)
Supplement: Supplementary file 3 — Reporting Summary [file 41467_2023_41037_MOESM3_ESM.pdf]

nature portfolio

Corresponding author(s): adrian.ranga@kuleuven.be, aabdelfattah@cemm.oeaw.ac.at

Last updated by author(s): Jul 28, 2023

Reporting Summary

Nature Portfolio wishes to improve the reproducibility of the work that we publish. This form provides structure for consistency and transparency in reporting. For further information on Nature Portfolio policies, see our Editorial Policies and the Editorial Policy Checklist.

Statistics

For all statistical analyses, confirm that the following items are present in the figure legend, table legend, main text, or Methods section.

n/a	Confirmed	
☐	☒	The exact sample size (*n*) for each experimental group/condition, given as a discrete number and unit of measurement
☐	☒	A statement on whether measurements were taken from distinct samples or whether the same sample was measured repeatedly
☐	☒	The statistical test(s) used AND whether they are one- or two-sided<br>*Only common tests should be described solely by name; describe more complex techniques in the Methods section.*
☒	☐	A description of all covariates tested
☐	☒	A description of any assumptions or corrections, such as tests of normality and adjustment for multiple comparisons
☐	☒	A full description of the statistical parameters including central tendency (e.g. means) or other basic estimates (e.g. regression coefficient) AND variation (e.g. standard deviation) or associated estimates of uncertainty (e.g. confidence intervals)
☐	☒	For null hypothesis testing, the test statistic (e.g. *F*, *t*, *r*) with confidence intervals, effect sizes, degrees of freedom and *P* value noted<br>*Give P values as exact values whenever suitable.*
☒	☐	For Bayesian analysis, information on the choice of priors and Markov chain Monte Carlo settings
☒	☐	For hierarchical and complex designs, identification of the appropriate level for tests and full reporting of outcomes
☐	☒	Estimates of effect sizes (e.g. Cohen's *d*, Pearson's *r*), indicating how they were calculated

Our web collection on statistics for biologists contains articles on many of the points above.

Software and code

Policy information about availability of computer code

Data collection	Organoid sizes, area ratios, nucleus distances from MagCs, sizes of MagCs were obtained through image evaluation on ImageJ (Version 1.52a). Magnetic field strengths were collected from Magnetic field simulation performed on Finite Element Magnetic Method software (FEMM 4.2).
Data analysis	GraphPad Prism 6 (Version 6.01), Microsoft® Excel® for Microsoft 365 MSO (Version 2212), corrcoef function MATLAB (R2018a, The MathWorks Ink.), R (version 4.1.0) package heatmap.2 using RStudio (RStudio Team (2020). RStudio: Integrated Development for R. RStudio, Inc., Boston, MA URL http://www.rstudio.com/)

For manuscripts utilizing custom algorithms or software that are central to the research but not yet described in published literature, software must be made available to editors and reviewers. We strongly encourage code deposition in a community repository (e.g. GitHub). See the Nature Portfolio guidelines for submitting code & software for further information.

Data

Policy information about availability of data

All manuscripts must include a data availability statement. This statement should provide the following information, where applicable:

- Accession codes, unique identifiers, or web links for publicly available datasets
- A description of any restrictions on data availability
- For clinical datasets or third party data, please ensure that the statement adheres to our policy

Source data is provided with this paper.

Human research participants

Policy information about studies involving human research participants and Sex and Gender in Research.

Reporting on sex and gender	not applicable
Population characteristics	not applicable
Recruitment	not applicable
Ethics oversight	not applicable

Note that full information on the approval of the study protocol must also be provided in the manuscript.

Field-specific reporting

Please select the one below that is the best fit for your research. If you are not sure, read the appropriate sections before making your selection.

☒ Life sciences ☐ Behavioural & social sciences ☐ Ecological, evolutionary & environmental sciences

For a reference copy of the document with all sections, see nature.com/documents/nr-reporting-summary-flat.pdf

Life sciences study design

All studies must disclose on these points even when the disclosure is negative.

Sample size	Experiments were conducted with a minimum of three independent biological replicate experiments. Except for the evaluation of the single lumen experiment (n = 2), and the EdU experiment (n = 2). The sequential steps to reach viable organoids at day 11 results in a consistent loss of organoids that can be analyzed. This was due to, overwhelming excess of MagCs during centrifugation in certain organoids, absence of MagCs within certain organoids, and organoids sinking to the culture plate-PEG hydrogel interface where they lose their organoid morphology. Each biological replicate can thus generally generate 10 to 15 analyzable magnetic organoids (considered technical replicates). To maximized the sample size, we considered all viable organoids in the study. We also relied on our previous experience to determine that this method is sufficient as it led to reproducible and similar results, this was also sufficient to emphasize differences between conditions such as during magnetic actuation and control conditions.
Data exclusions	Magnetoids that lost their morphology due to their proximity to the well floor, or the hydrogel-medium interface were not considered in the analysis.
Replication	Data was replicated using a minimum of two and a maximum of 4 independent experiments to ensure results were reproducible. All replicates were successful except for the third replicate of the control condition in the magnetic attraction experiment and the fourth replicate in the PAX6 expression "No Mag" experiment due to contamination. The experiment was repeated again which is why the "control" and "Mag" conditions in the PAX6 expression experiment are 5 replicates each, while the "No Mag" experiment has 4 replicates.
Randomization	Starting and culture conditions were kept constant to eliminate sample-specific characteristics. A source of randomness can arise from the location of MagCs in the organoids and the position of the magnetoids withing the PEG hydrogel which can affect the applied magnetic force as this will change the distance between the magnet surface and the magnetoid. Nevertheless, given the reproducibility of the observations we believe that the observed results are due to the experimental conditions (biochemical and/or mechanical).
Blinding	Investigators were not blinded to group allocation. The metric of patterning relied on an equation to limit investigator bias.

Reporting for specific materials, systems and methods

We require information from authors about some types of materials, experimental systems and methods used in many studies. Here, indicate whether each material, system or method listed is relevant to your study. If you are not sure if a list item applies to your research, read the appropriate section before selecting a response.

Materials & experimental systems

n/a	Involved in the study
☐	☒ Antibodies
☐	☒ Eukaryotic cell lines
☒	☐ Palaeontology and archaeology
☒	☐ Animals and other organisms
☒	☐ Clinical data
☒	☐ Dual use research of concern

Methods

n/a	Involved in the study
☒	☐ ChIP-seq
☒	☐ Flow cytometry
☒	☐ MRI-based neuroimaging

Antibodies

Antibodies used

FOXA2, Santacruz (sc-374376), mouse monoclonal, dilution 1:200
PAX6, Biolegend (901301), rabbit polyclonal, dilution, 1:200
NKX2.2, DSHB (DSHB - 74.5A5), mouse monoclonal, dilution 1:200
OLIG2, R&D systems (AF 2418), goat polyclonal, dilution 1:200
NKX6.1, DSHB (DSHB - F55A10), mouse monoclonal, dilution 1:200
ISL1/2, DSHB (DSHB - 39.4D5), mouse monoclonal, dilution 1:200
TUBB3, Biolegend (802001), rabbit polyclonal, dilution 1:200

Alexa Fluor 555, Invitrogen (A-31570), donkey polyclonal, donkey anti mouse, dilution 1:500
Alexa Fluor 647, Invitrogen (A-31573), donkey polyclonal, donkey anti rabbit, dilution 1:500
Alexa Fluor 647, Invitrogen (A-21447), donkey polyclonal, donkey anti goat, dilution 1:500

Validation

FOXA2, Santacruz (sc-374376), mouse monoclonal, dilution 1:200
Wulansari et al., Science Advances 7(8):eabb1540, February 17, 2021

PAX6, Biolegend (901301)
Quadrato et al., Nature 545(7652):48–53, May 4, 2017

NNKX2.2, DSHB (DSHB - 74.5A5)
Luisier et al., Nature Communications 9:2010, May 22, 2018

OLIG2, R&D systems (AF 2418), NKX6.1, DSHB (DSHB - F55A10) and ISL1/2, DSHB (DSHB - 39.4D5)
Ranga et al., PNAS 113(44):E6831-E6839, October 14, 2016

TUBB3, Biolegend (802001)
Yissachar et al., 2017, Cell 168:1135–1148 March 9, 2017

Eukaryotic cell lines

Policy information about cell lines and Sex and Gender in Research

Cell line source(s)

NCRM-1 (RRID:CVCL_1E71) hPSC line from NIH Center for Regenerative Medicine (CRM), Bethesda, USA.
ZO1 hPSC line (Mono-allelic mEGFP-Tagged TJP1 WTC, Coriell institute for Medical Research)
hPSC F-actin reporter line provided by Catherine Verfaillie, Stem Cell institute Leuven.

Authentication

All hIPS cell line have been authenticated by the original sources or internally by immunostaining for pluripotency markers.

Mycoplasma contamination

All cell lines tested negative for mycoplasma contamination

Commonly misidentified lines
(See ICLAC register)

No commonly misidentified cell lines listed by ICLAC were used in this work
